# Development of a Bispecific Antibody-Based Platform for Retargeting of Capsid Modified AAV Vectors

**DOI:** 10.3390/ijms22158355

**Published:** 2021-08-03

**Authors:** Juliane Kuklik, Stefan Michelfelder, Felix Schiele, Sebastian Kreuz, Thorsten Lamla, Philipp Müller, John E. Park

**Affiliations:** 1 Division of Cancer Immunology and Immune Modulation, Boehringer Ingelheim Pharma GmbH & Co. KG, 88387 Biberach an der Riss, Germany; juliane.kuklik@boehringer-ingelheim.com; 2 Division of Research Beyond Borders, Boehringer Ingelheim Pharma GmbH & Co. KG, 88387 Biberach an der Riss, Germany; stefan.michelfelder@boehringer-ingelheim.com (S.M.); sebastian.kreuz@boehringer-ingelheim.com (S.K.); 3 Division of Biotherapeutics Discovery, Boehringer Ingelheim Pharma GmbH & Co. KG, 88387 Biberach an der Riss, Germany; felix.schiele@boehringer-ingelheim.com; 4 Boehringer Ingelheim Venture Fund GmbH, 55216 Ingelheim am Rhein, Germany; philipp_3.mueller@boehringer-ingelheim.com; 5 Division of Drug Discovery Sciences Biberach, Boehringer Ingelheim Pharma GmbH & Co. KG, 88387 Biberach an der Riss, Germany; thorsten.lamla@boehringer-ingelheim.com

**Keywords:** adeno-associated viral vectors, AAV, protein engineering, retargeting, bispecific antibody, capsid modification, FAP, PD-L1

## Abstract

A major limiting factor for systemically delivered gene therapies is the lack of novel tissue specific AAV (Adeno-associated virus) derived vectors. Bispecific antibodies can be used to redirect AAVs to specific target receptors. Here, we demonstrate that the insertion of a short linear epitope “2E3” derived from human proprotein-convertase subtilisin/kexin type 9 (PCSK9) into different surface loops of the VP capsid proteins can be used for AAV de-targeting from its natural receptor(s), combined with a bispecific antibody-mediated retargeting. We chose to target a set of distinct disease relevant membrane proteins—fibroblast activation protein (FAP), which is upregulated on activated fibroblasts within the tumor stroma and in fibrotic tissues, as well as programmed death-ligand 1 (PD-L1), which is strongly upregulated in many cancers. Upon incubation with a bispecific antibody recognizing the 2E3 epitope and FAP or PD-L1, the bispecific antibody/rAAV complex was able to selectively transduce receptor positive cells. In summary, we developed a novel, rationally designed vector retargeting platform that can target AAVs to a new set of cellular receptors in a modular fashion. This versatile platform may serve as a valuable tool to investigate the role of disease relevant cell types and basis for novel gene therapy approaches.

## 1. Introduction

Recombinant adeno-associated virus (rAAV)-based vector systems became popular in recent years as vehicles for gene therapy approaches, such as the well described serotype 2 [1,2]. AAVs belong to the family of *Parvoviridae* and are non-pathogenic with a very good safety profile, are unrelated to any human disease [3,4], and are capable of providing stable gene expression over an extended period of time [1,5]. The virus has an approximate diameter of 22 nm, is non-enveloped, and has a packaging capacity of up to 4.7 kb [6,7,8]. The AAV capsid is composed of the assembly-activating protein (AAP) [9] and 60 viral proteins VP1, VP2, and VP3 with a ratio of 1:1:10 [10,11,12]. During AAV2 infection, first, cellular contact is made via heparin sulfate–proteoglycan (HSPG) interaction, which mainly involves five basic amino acids (R484, R487, K532, R585, and R588) [13,14,15]. Following cell attachment, AAV2 internalizes upon co-receptor engagement. A well-known co-receptor is integrin α5β1 that interacts with the conserved NGR motif (511–513) [13,16]. Additional known co-receptors include hepatocyte growth factor receptor [17], CD9 [18], fibroblast growth factor recptor-1 [19], laminin receptor [20], and GPR108 [21]. The wide range of potential co-receptors results in a broad host tropism. AAV2 transduces a wide variety of dividing and non-dividing cells in vitro and in vivo such as muscle, liver, brain, lung, and tumor tissue [22,23,24,25,26,27,28,29,30]. In contrast, many therapeutic approaches to diseases such as cancer aim to restrict the activity of the therapy to the diseased area or to cells expressing certain cell surface antigens to avoid side effects in normal tissues [31]. The lack of capsids selective for disease-relevant cell surface markers and broad tropism of AAV limits the usability of AAVs as a systemically applied therapeutic in this context. Finally, broad infection of diverse tissues necessarily dilutes viral genomes and reduces the optimal transduction of the desired tissue. Unnecessarily high vector doses or widespread capsid, transgene distribution, and therapeutic payload expressed in non-relevant (“off-target”) tissues may cause activation of the immune system (e.g., T-cell activation via TLR9) [32], acute decline in platelets, complement activation, or even serious adverse events including acute hepatotoxicity [33]. A highly selective retargeting AAV would be expected to require much lower doses and be more selectively distributed.

Several targeting technologies were developed that aim to (re)direct the AAV capsid to cell-type specific receptors [2,34]. “Detargeting” in the context of AAV refers to modification of capsids in a manner which eliminates the natural capsid–receptor interactions that allow the natural viral infection. The term “retargeting” describes generation of novel capsids which specifically interact with the desired cell surface receptor(s) of interest and enable transduction of cells bearing those receptors. AAV detargeting can be achieved by capsid modification with exogenous agents such as HPMA polymers, PEGylation, or cationic lipid coating [35,36,37]. This does not require capsid protein modification and offers the additional benefit of shielding the capsid from antibody neutralization, however, it does not allow for receptor targeting. One of the first retargeting approaches used bispecific antibodies directly binding to the unmodified AAV capsid and platelet-specific αIIbβ3 integrin to enhance cell type selective transduction [38]. However, this approach does not impede the natural viral tropism. Simultaneous detargeting and retargeting to alternative receptors further requires capsid modifications on the protein level. It was shown that AAV capsids tolerate amino acid modifications [39,40] and insertions, which are preferentially inserted within the variable loop eight at capsid position 587/588 [10,11]. Small ligands, nanobodies, or DARPins that directly bind target receptors were successfully inserted into the AAV capsid, e.g., by genetic fusion or chemical ligation to a capsid protein [41,42,43,44,45,46].

Enabling direct and selective binding of a desired receptor via AAV re-engineering, if feasible, requires a de-novo capsid development for every new target [2,47,48], which, even if successful, does not always provide mechanistic insight into the molecular targets engaged, nor does it assure that such targeting works across species [49]. In contrast, the insertion of a single peptide epitope recognized by a unique monoclonal antibody into the AAV capsid may result in AAV de-targeting combined with the option of bispecific adaptor-mediated retargeting. Such an approach is highly attractive, modular, and flexible with regard to target selection, as the exchange of only one arm of the adaptor allows for the retargeting to different receptors without the need to further modify the engineered AAV.

In this study, we generated novel AAV2-based rAAV variants by insertion of a short peptide epitope derived from an alpha helical region of proprotein-convertase subtilisin/kexin type 9 (PCSK9) into different regions of the AAV2 surface. Recognition of the inserted peptide was facilitated by an antibody which bound the linear epitope spanning from Ile161 to Glu170 (ITPPRYRADE, KD = 4.96 nM). This epitope (2E3) was identified by peptide microarrays, and essential amino acids for antibody binding were identified by surface plasmon resonance peptide analysis and crystallography-based protein structure determination. The epitope can be shortened to eight amino acids (TPPRYRAD, KD = 22.1 nM) with only a modest loss of affinity [50].

We established a modular system to target the receptors fibroblast activation protein (FAP) and programmed death-ligand 1 (PD-L1). Both markers are disease relevant membrane proteins [51,52,53,54,55] and neither were previously described as AAV-gene therapy targets. Both receptors are expressed by tumors or within their microenvironment of which cancer-associated fibroblasts are a prominent cell type [56,57,58,59,60,61,62]. FAP is an integral membrane bound glycoprotein with serine protease, post-proline dipeptidyl peptidase, and endopeptidase enzymatic activity [63,64]. It is highly conserved between human and mice [63] and expressed in reactive stromal fibroblasts of epithelial neoplasms as well as fibrosis [65]. Normally, the expression is upregulated in fetal mesenchymal tissue and downregulated in adult tissues [66]; exceptions are cancer, wound healing, and cell migration of bone marrow mesenchymal stem cells [67,68,69]. The increased expression of FAP in the tumor stroma and its absence from healthy adult tissues make this marker an especially attractive target for anti-cancer gene therapies [70]. FAP was described to be successfully targeted by antibodies [71], anti-FAP fab-coated immunoliposomes [72], and T-cell engagers [73], but an AAV based gene therapy targeting FAP as a novel cell entry receptor was not described yet.

PD-L1 is a 33 kDa type I transmembrane protein of the immuno-globulin superfamily and one ligand of programmed cell death protein 1. The interaction with the latter helps to maintain immune tolerance [74,75,76]. High mRNA expression levels of PD-L1 can be found in heart, skeletal muscle, placenta, and lung tissues, and low-level expression can be found in thymus, spleen, kidney, and liver tissues [74]. Upon interferon gamma stimulation, PD-L1 is expressed by monocytes and keratinocytes [75]. PD-L1 expression was described in many tumors and their microenvironments [51,60,61,62,77] and is a main component of the tumor’s armamentarium to prevent immune destruction by inhibition of activation, expansion, and effector functions of T-cells [75,78,79]. To overcome the immune suppressive environment of PD-L1 expressing tumor cells, several antibodies were approved blocking either PD1 or PD-L1 [54]. Furthermore, downregulation of PD-L1 expression levels by siRNA in lipidoid nanoparticles resulted in upregulated immune cell activation [80]. However, targeting of PD-L1 positive cells was not yet described as a gene therapy approach.

As FAP and PD-L1 are abundantly expressed on tumor cells and within the tumor microenvironment, our approach has the potential to target a significant portion of human cancers using the same rAAV variant in combination with different bispecific antibodies. Accessibility of the tumor microenvironment for antibodies via stromal blood vessels was described previously, and tumor stromal cells show a higher genetic stability and less turnover than tumor cells [66,81]. FAP as well as PD-L1 were described to internalize upon antibody binding, and numerous antibodies binding those receptors were developed and are readily available [59,71,81,82,83,84]. Therefore, we hypothesized that antibody armed AAVs are readily taken up by cells upon FAP or PD-L1 binding.

We demonstrate that an epitope insertion successfully eliminated the AAV’s natural tropism and can serve as an anchor for novel retargeting of bispecific antibodies. Bispecific antibody–rAAV-2E3 complexes were able to successfully target FAP and PD-L1 expressing cells for the first time in vitro.

## 2. Results

### 2.1. Design and Characterization of AAV2 Particles with Novel Epitope Insertion Domains

**Figure 1:**
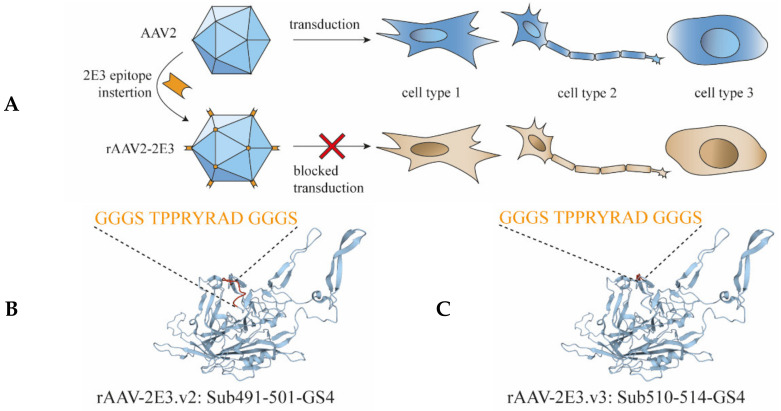

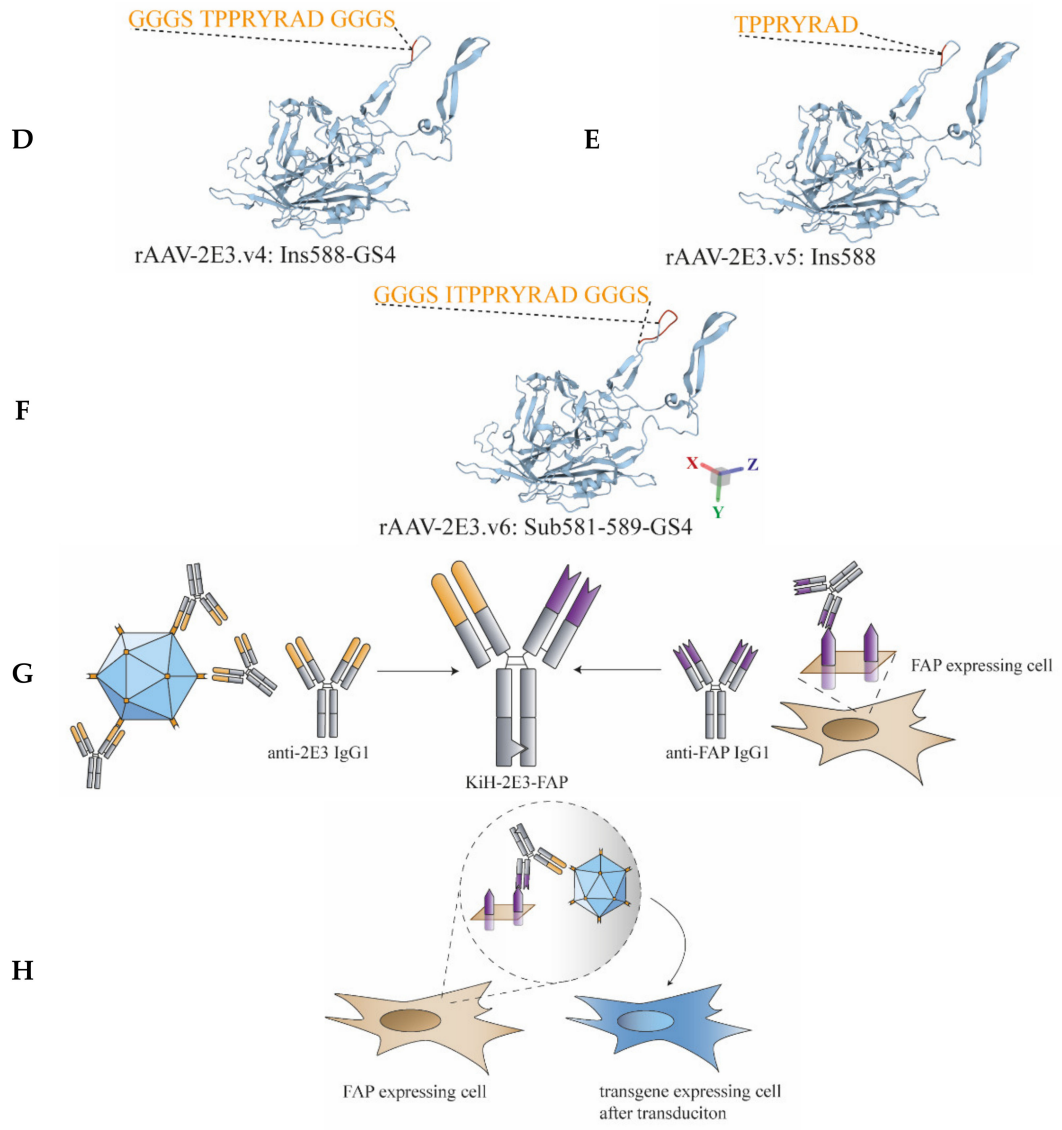
Design of novel AAV capsids and bispecific antibodies to develop a retargeting platform. (**A**) Five versions of linear 2E3 epitopes (orange) were inserted into different areas of the AAV2 capsid, resulting in five novel rAAV-2E3 capsid variants. The inserted peptide aims for the destruction of natural AAV2 tropism and should result in novel rAAV-2E3 variants with reduced transduction properties. Transduced cells are shown in blue, and non-transduced cells are shown in beige. (**B**–**F**) Ribbon drawing of the VP3 AAV2 subunit. Wild-type structures are shown in blue, and modified regions are indicated in red. Epitope sequences that were inserted and resulted in rAAV-2E3.v1-6 are shown in orange letters. Structures were derived and visualized using the PDB online tool (https://.rcsb.org; Access date: 22.05.2021) based on ID 1LP3. (**G**) Illustration of the design of a knob-into-hole bispecific antibody derived from engineering of monospecific anti-2E3 epitope binding antibody (orange) and a monospecific anti-FAP binding antibody (violet). (**H**) Schematic illustration of the established re-targeting mechanism. A complex of novel rAAV-2E3 variant was formed with bispecific antibody KiH-2E3-FAP. The bispecific antibody served as adaptor between 2E3 epitope and cell surface receptor FAP. Cells expressing FAP were bound, resulting in transduction and gene expression (blue cell).

**Figure 2:**
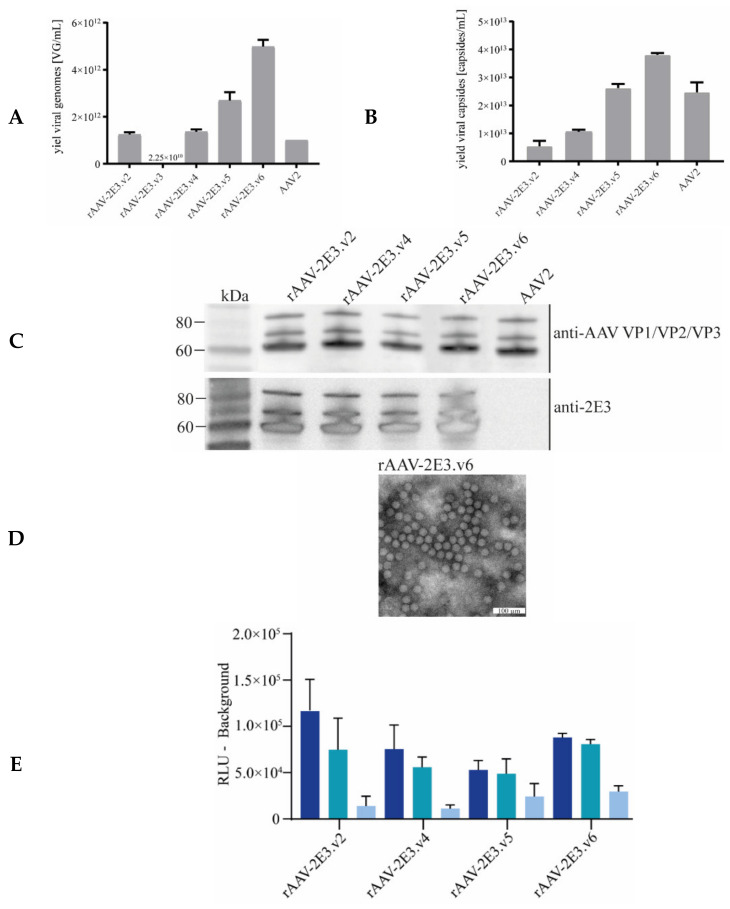
Production and characterization of rAAV-2E3 capsid variants. (**A**) Packaging efficiency of purified rAAV-2E3 particles (v2, v3, v4, v5, and v6) and AAV2. Experiments were performed in biological triplicates of each batch, and data are represented as mean + SD. (**B**) The vector yield of rAAV-2E3 variants and AAV2 was measured by ELISA. Experiments were performed in biological triplicates of each batch, and data are represented as mean ± SD. (**C**) Western blot and staining of reduced rAAV-2E3 variants and AAV2 with anti-VP antibody B1 (Progen, Heidelberg, Germany) or anti-2E3 antibody showing the presence of VP1:VP2:VP3 proteins and the insertion of the 2E3 epitope in all VP proteins of modified capsid variants. (**D**) Transmission electron microscopy of negative stained rAAV-2E3.v6 viral particles. Scalebar 100 µm. (**E**) Immobilization of rAAV-2E3 serotypes and AAV2 following binding of anti-2E3 antibody in serial dilutions (1:1000 dark blue, 1:10,000 turquoise, and 1:100,000 light blue) and detection via Sulfo-tag labeled anti-human IgG in a MSD^®^-ECL ELISA assay. Data represent mean + SD of three independent experiments.

In order to detarget and, at the same time, render AAV2 capsids amendable for binding by antibodies, a panel of short peptide sequences (2E3) of 9 to 17 amino acids (including Gly-Ser linkers) derived from PCSK9 were inserted at various capsid sites that are believed crucial for natural transduction of cells (Table 1; Figure 1A–F). The viral capsid position 491 to 501 (VP1 numbering) was substituted with our GS-linker framed 2E3 sequence (rAAV-2E3.v2) or substituted at position 510–514 (rAAV-2E3.v3) to alter the conserved NGR sequence (511–513) responsible for integrin α5β1 binding. Furthermore, 2E3 was inserted at position 588 with and without GS-linkers (rAAV-2E3.v4 and rAAV-2E3.v5), and the position 581 to 589 was substituted with 2E3 (rAAV-2E3.v6). These positions were chosen to ablate the HSPG binding motive at position 585 and 588. The rAAV-2E3 variants and the wild-type AAV2 encoding GFP under the CMV promoter were produced separately in HEK 293-H cells. Following rAAV2 purification, the packaging efficiency (VG/mL) and the vector yield (capsids/mL) were measured by ddPCR and ELISA. Except for rAAV-2E3.v3 (2.25 × 10^10^ VG/mL), all engineered capsid variants yielded comparable titers ranging from 1.24 × 10^12^ to 4.98 × 10^12^ VG/mL with good correlation to viral capsid yield (Figure 2A,B). The purified viral capsids were analyzed by western blot. Detection of VP1, VP2, and VP3 proteins showed the expected capsid protein ratio of 1:1:10. The inserted 2E3 epitope could be readily detected by antibody staining in all three VP proteins. Therefore, we concluded that 60 2E3 epitopes were exposed on the rAAV-2E3 capsid surface (Figure 2C). EM images were taken of rAAV2-2E3.v6 showing the characteristically icosahedral AAV capsid structure and overall a high packaging efficiency (exemplary image of rAAV-2E3.v6 shown in Figure 2D, uncropped Figure S1). To permit successful bispecific antibody retargeting, the inserted epitope in our rAAV-2E3 capsids must be accessible to antibody binding. Therefore, we measured the 2E3-antibody binding of immobilized capsid variants in an MSD^®^-ECL ELISA assay. All produced rAAV-2E3 capsid variants as well as AAV2 could be detected by anti-AAV2 polyclonal antibodies, and all rAAV-2E3 variants were detected with our monoclonal anti-2E3 antibody (Figure 2E and Figure S2).

### 2.2. Novel rAAV-2E3 Variants Show Altered Transduction and Binding Properties to HSPG Compared to AAV2

**Figure 3:**
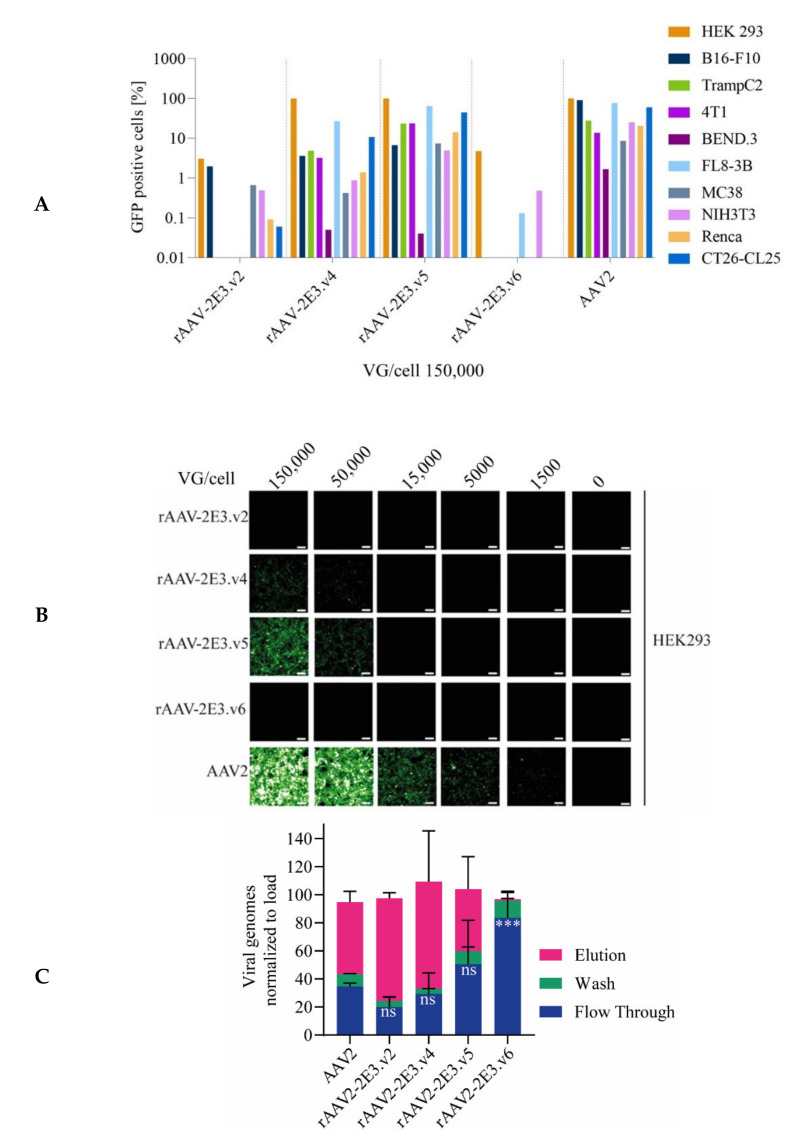
Infectivity and heparin binding differed amongst rAAV-2E3 variants. (**A**) Screen of infectivity of rAAV-2E3 capsid variants in comparison to AAV2 was performed on selected human and murine cell lines. Viral particles were incubated with cells (VG/cell 150,000) for three days followed by flow cytometry measurement of GFP expression. (**B**) HEK 293 cells infected with decreasing VG/cell (150,000, 50,000, 15,000, and 5000) of AAV2 in comparison to rAAV-2E3 capsid variants. Images of GFP expression were taken after 3 days of infection (scalebar 100 µm). (**C**) Analysis of rAAV-2E3 serotype interactions with heparin-agarose in comparison to AAV2. Equal amounts of rAAV-2E3 particles were loaded to heparin columns, fractions of flow through, wash, and elution were collected, and viral genomes were measured by ddPCR. The data were normalized versus the total amount of loaded viral genomes. Data represent the mean + SEM of two independent experiments. One-way ANOVA compared to AAV2 flow through, uncorrected Fisher’s LSD, *** *p* < 0.001, ns = non-significant.

The impact of 2E3 epitope insertions on cellular transduction was analyzed for all AAV-2E3 variants and compared to AAV2 transduction on a panel of human and murine cell lines. As expected, we detected a strongly reduced GFP expression for rAAV-2E3.v2 and rAAV-2E3.v6, even at a high levels of viral genomes/cell (VG/cell) of 150,000 (Figure 3A,B) for all cell lines compared to AAV2 mediated GFP expression within the same cells. Both rAAV-2E3 variants presented cellular detargeting independent of the location of the 2E3 epitope substitution (Figure 1B,F), suggesting that the altered capsids at positions 491 and 581 strongly interfered with the ability of rAAV to efficiently transduce cells in vitro. Cells transduced with variants rAAV-2E3.v4 and rAAV-2E3.v5 showed GFP expression on several cell lines but to a lesser extent compared to the parental AAV2 (Figure 3A,B). These variants had a 2E3 insertion at position 588, and v5 differed in the use of a GS-linker compared to v4 (Figure 1D,E).

AAV2 infection is initiated by virus attachment to HSPG [13]. To further investigate the effects of 2E3 insertion on cellular binding, we mimicked this interaction by using heparin columns [15,85,86]. About two thirds of wild type AAV2 particles bound to the heparin column, and binding could be efficiently reverted using 2M NaCl elution buffer. Variants rAAV-2E3.v2 to .v5 retained heparin interaction and release comparable to AAV2. In contrast, rAAV-2E3.v6 showed significantly reduced heparin binding compared to AAV2, since nearly all viruses could be found in the flow through (mean 83.5% ± 27.05%, *p* < 0.001) and wash fractions (Figure 3C). These experiments confirmed that site-specific insertion of the 2E3 epitope can interfere with cellular HSPG binding of rAAV-2E3 variants, thereby leading to a significant decrease in cell transduction efficiency.

### 2.3. Engineering and Characterization of Bispecific Antibodies Recognizing 2E3 Epitope and Human FAP Receptor

**Figure 4:**
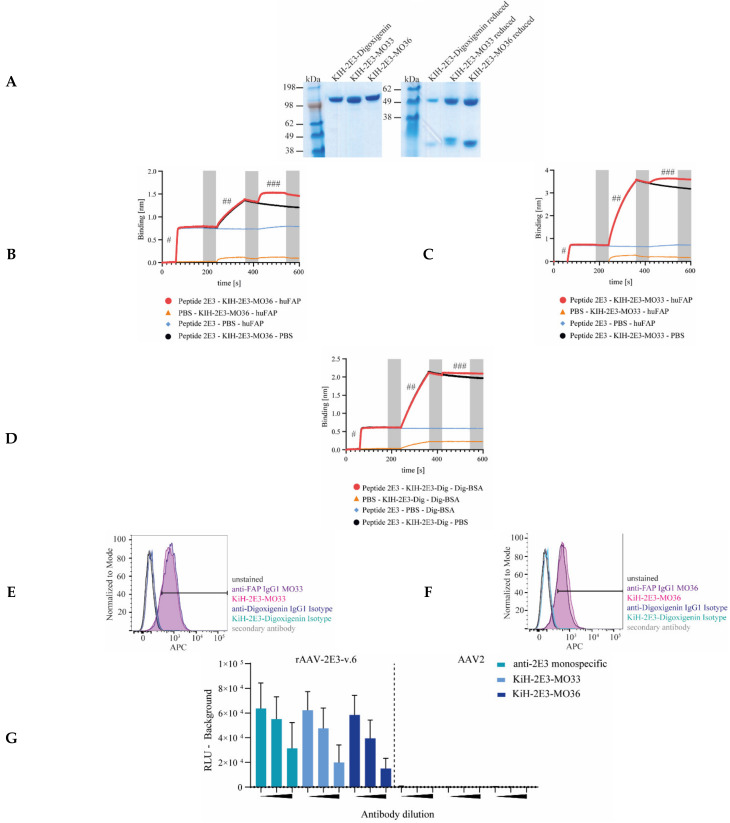

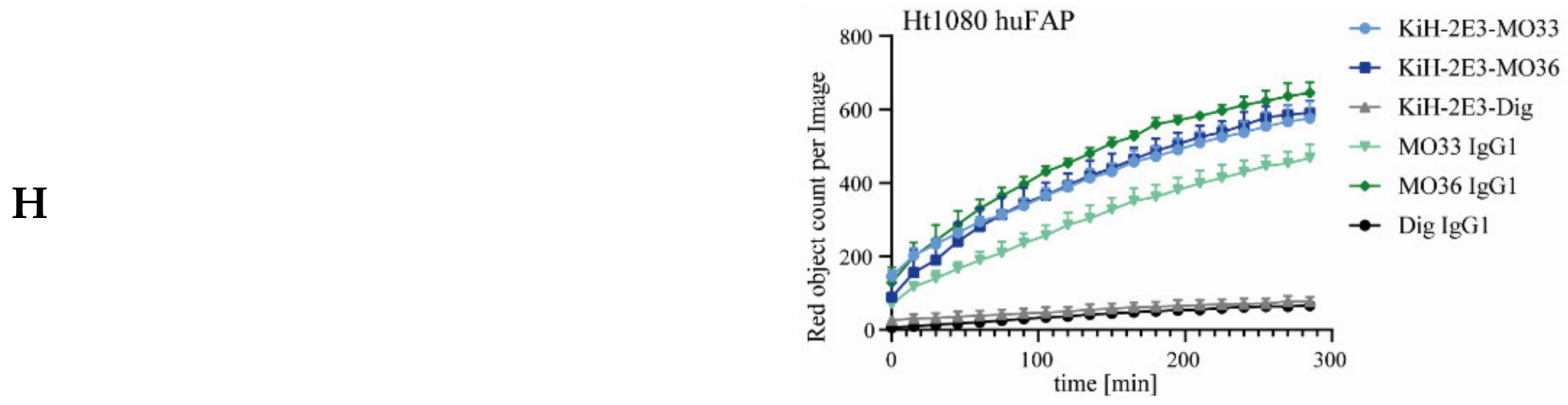
Production and characterization of bispecific antibodies binding both the cell surface receptor FAP and the 2E3 epitope. (**A**) Purified KiH-2E3-MO33, KiH-2E3-MO36, and KiH-2E3-digoxigenin production analysis under reducing and non-reducing conditions by SDS page and protein staining. (**B**–**D**) Bridging assay proved the simultaneous binding of 2E3 epitopes and target proteins by KiH-bispecific antibodies. Biotin-labeled 2E3 peptides bound streptavidin tips and resulted in first spectral shift (#), (**B**) KiH-2E3-MO33, (**C**) KiH-2E3-MO36, and (**D**) KiH-2E3-dig interaction with the 2E3 epitope resulted in second spectral shift (##), and interaction with recombinant FAP or BSA-digoxigenin resulted in third spectral shift (###). Wash steps are indicated by grey bars. The novel KiH antibodies were analyzed for FAP binding in comparison to the original (**E**) MO33 and (**F**) MO36 anti-FAP antibodies by flow cytometry staining. (**G**) Comparison of KiH-antibody and anti-2E3 IgG antibody binding of rAAV-2E3.v6 and AAV2 immobilized capsids. Antibody dilutions (1:1000, 1:10,000, and 1:100,000) are indicated by black triangles, and light signals were detected after Sulfo-tag labeled anti-human IgG incubation by MSD^®^-ECL. Data show mean ± SD of three independent experiments. (**H**) Human Fabfluor-pH Red Antibody Labeling Dye coupled with bispecific and monospecific antibodies resulted in red fluorescence signals after internalization by HT1080 huFAP cells. Data show mean + SD of two independent experiments.

Bispecific knob-into-holes (KiH) antibodies were designed and produced according to previously described procedures [87] and were used to arm the rAAV-2E3 capsids to function as an adapter to FAP expressed on the target cells. A CH3 mutation (K409R) was generated to form a “knob” in the Fc-region of the anti-2E3 IgG1, and the complementary “hole” CH3 mutation (K409L) was similarly created in the Fc-region of the clone MO33 as well as the clone MO36-derived anti-FAP IgG1 antibodies (Figure 1G). Furthermore, the mutations L243A and L235A were inserted into the CH2 domain of all antibodies to eliminate Fc-mediated effector functions [88]. Bispecific antibodies (KiH-2E3-MO33, KiH-2E3-MO36, and isotype control KiH-2E3-digoxigenin) underwent three purification steps (protein A, cation exchange, and size exclusion chromatography). Purified proteins are shown in Figure 4A. To prove that bispecific antibodies were produced and not a mix of monospecific antibodies, we performed a bridging assay using biolayer light interferometry to monitor simultaneous binding of 2E3 and FAP or digoxigenin epitopes. First, biotinylated 2E3-peptides were coupled to streptavidin sensor tip (first spectral shift, indicated by #); binding of KiH-bispecific antibodies resulted in a second spectral shift (##), and final interaction with soluble FAP or BSA-labeled digoxigenin resulted in a third spectral shift (###) (Figure 4B–D). Bispecific antibodies were analyzed to interrogate 2E3 epitope and FAP binding compared to the original monospecific antibodies. To this end, flow cytometry staining and analysis of HT1080 huFAP expressing cells with monospecific IgGs MO33, MO36, bispecific KiH-2E3-MO33, KiH-2E3-MO36, and respective isotype controls were performed. A clear shift of all anti-FAP reagents (MO33: 87.4%; KiH-2E3-MO33: 85.5%; MO36: 79.2%, and KiH-2E3-MO36: 81.8% APC positive cells) relative to the corresponding isotype controls was demonstrated. Overlaying shifts of FAP positive cells, monospecific IgG MO33 compared to bispecific KiH-2E3-MO33, and monospecific IgG MO36 compared to bispecific KiH-2E3-MO36 indicated no loss of FAP binding (Figure 4E,F). All bispecific antibodies bound to immobilized AAV-2E3.v6 capsids, comparable to the monospecific 2E3 antibody, and none interacted with wild type AAV2 capsids (Figure 4G). Immobilization of AAV-2E3.v6 and AAV2 was proven by incubation with anti-AAV2 antibody A20 (Progen, Heidelberg, Germany) and respective secondary antibody anti Mouse Antibody Goat SULFO-TAG (MSD^®^). Specific binding of the secondary antibody was compared to the isotype control anti Human Antibody Goat SULFO-TAG (MSD^®^) (Figure S3). FAP is known to internalize upon antibody binding [72,81]. Whether these bispecific antibodies could induce FAP internalization was analyzed using HT1080 huFAP expressing cells. The bispecific and the monospecific antibodies were Fc-conjugated with a pH sensitive dye, emitting red fluorescence at low pH. Therefore, red fluorescence signals were associated with antibody internalization into endosomes. Cells incubated with labeled MO36 IgG1, MO33 IgG1, KiH-2E3-MO36, and KiH-2E3-MO33 showed increasing red fluorescence signals with a clear shift as compared to the corresponding isotype control (Figure 4H). This indicates that FAP internalization can be induced by binding of (bispecific) antibodies.

All in all, we generated bispecific antibodies able to induce FAP internalization and simultaneously engage FAP and 2E3 epitopes without losing specificity in comparison to parental monospecific antibodies.

### 2.4. Successful Retargeting of Novel rAAV-2E3 Capsids in FAP Expressing Cells via Bispecific Antibodies

**Figure 5:**
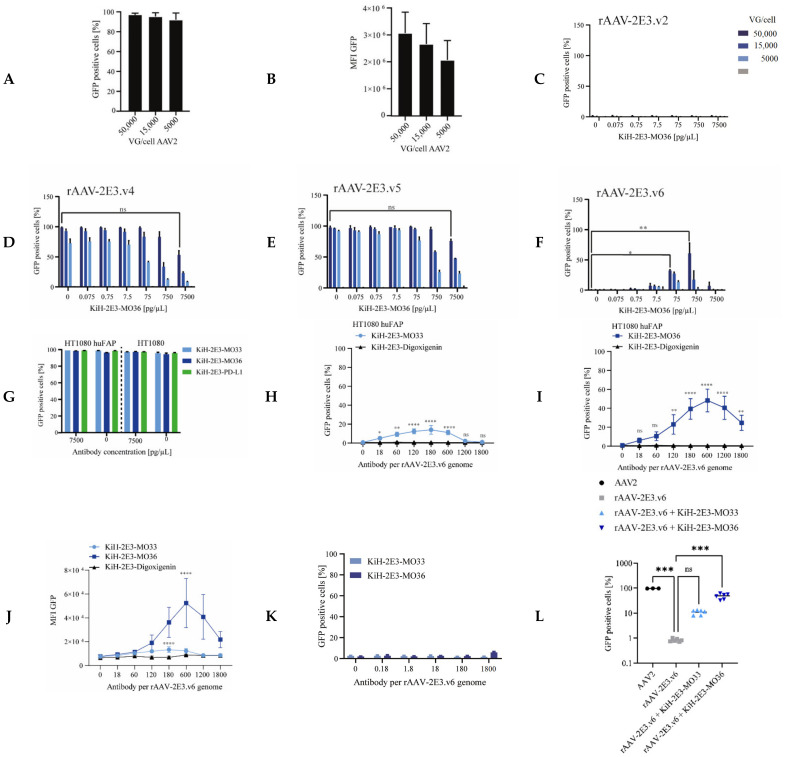
Retargeting novel rAAV-2E3 to FAP expressing cells using KiH bispecific antibodies. HT1080 huFAP cells were treated with AAV viral variants for 3 days following flow cytometry analysis. (**A**) AAV2 transduction with titrated VG/cell and analysis of GFP positive proportion and (**B**) MFI levels. Data show mean + SD. (**C**) Proportion of cellular GFP expression after retargeting with rAAV-2E3.v2, (**D**) rAAV-2E3.v4, (**E**) rAAV-2E3.v5, and (**F**) rAAV-2E3.v6 complexed with KiH-2E3-MO36. (**D**–**F**) share legend shown in (**C**). Data show mean + SD of two independent experiments, one-way ANOVA, uncorrected Dunn’s test, * *p* < 0.05; ** *p* < 0.01, ns= non-significant. (**G**) Bispecific antibodies pre-incubated with AAV capsids did not affect GFP expression levels of transduced cells. Data show mean + SD. (**H**) Titration of KiH-2E3-MO33, (**I**) KiH-2E3-MO36, and isotype control rAAV-2E3.v6 (VG/cell 50,000) and analysis of percentage of GFP expressing cells and (**J**) MFI levels. Data show mean + SD of three independent experiments, one-way ANOVA, uncorrected Dunn’s test groups compared 0.0 pg/µL KiH-2E3, * *p* < 0.05; ** *p* < 0.01; **** *p* < 0.0001, ns = non-significant. (**K**) rAAV-2E3.v6 complexed with KiH-2E3-MO33 or KiH-2E3-MO36 and incubation on HT1080 huFAP negative cells did not result in GFP expression. Data show mean + SD. (**L**) Direct comparison of HT1080 huFAP GFP expression levels after transduction with AAV2, rAAV-2E3.v6, rAAV-2E3.v6:KiH-2E3-MO33, and rAAV-2E3.v6:KiH-2E3-MO36 at 50,000 VG/cell and 2.5 ng/µL bispecific antibody, one-way ANOVA, uncorrected Dunn’s test *** *p* < 0.001, ns = non-significant.

Purified rAAV-2E3 variants were pre-incubated with bispecific KiH-2E3-FAP antibodies to form a FAP targeting complex. AAV internalization via FAP binding should result in viral uptake and finally viral transgene expression in FAP expressing cells, assuming the antibody complex does not interfere with uncoating of rAAV-2E3 or cytoplasmic release (Figure 1H). The ability to retarget novel rAAV-2E3 capsids to HT1080 huFAP expressing cells was examined by titration of rAAV-2E3 variants at 50,000 to 5000 VG/cell against dilutions of bispecific KiH-2E3-MO36 antibodies. HT1080 huFAP incubation with our positive control AAV2 (VG/cell 50,000, 15,000, and 5000) proved successful transduction of HT1080 huFAP cells and resulted in up to 97% GFP expressing cells with a decrease of MFI with less VG per cell (Figure 5A,B). Variant rAAV-2E3.v2 showed no transduction at any antibody concentration (Figure 5C). Both variants rAAV-2E3.v4 and rAAV-2E3.v5 showed high transgene expression (up to 99% GFP positive cells) even without bispecific antibody binding and a trend to decreased GFP expression at the highest bispecific antibody levels. An additive retargeting effect within such high background expression could not be detected (Figure 5D,E). rAAV-2E3.v6 showed no GFP expression without bispecific antibody pre-incubation but significantly increased GFP expression at defined antibody per viral genome ratios (VG/cell 50,000 rAAV-2E3.v6 ± 75 pg/µL KiH-2E3-MO36: 32.29% ± 1.62% GFP positive cells; *p* < 0,05 and VG/cell 50,000 rAAV-2E3.v6 ± 750 pg/µL KiH-2E3-MO36: 60.8% ± 17.32% GFP positive cells; *p* < 0.01) (Figure 5F). Pre-incubation of the parental AAV2 with high amounts of the bispecific antibody (7500 pg/µL) did not affect AAV2 transduction of HT1080 cells lines (Figure 5G).

As might be expected, successful retargeting requires an optimal antibody to capsid ratio depending on the capsid variant and the bispecific antibody used. A ratio of 180 KiH-2E3-MO33 antibodies per rAAV-2E3.v6 genome resulted in a significant increase of GFP positive cells (mean percent of GFP positive cells 13.99 ± 4.23; *p* < 0.0001) (Figure 5H). Significantly higher amounts of GFP positive cells were obtained at a ratio of 600 KiH-2E3-MO36 antibodies per rAAV-2E3.v6 genomes (mean percent of GFP positive cells 48.28 ± 10.93; *p* < 0.0001) (Figure 5I). Increasing amounts of GFP positive cells also corresponded to an increase of MFI (Figure 5J). Low antibody ratios resulted in low transduction efficiencies, and overly high antibody ratios also reduced transduction efficiency of cells dramatically. Overload of bispecific antibodies possibly resulted in huge complexes too big for internalization or FAP receptor binding and blockage by free antibodies, as uncoupled antibodies were not depleted from the preparations used to transduce target cells. Furthermore, retargeting was shown to be dependent on FAP receptor expression, since HT1080 FAP negative cells could not be transduced by this retargeting approach (Figure 5K). Consistent with this, retargeting was absent when using a bispecific isotype control antibody composed of the same anti-2E3 arm paired with an anti-digoxigenin isotype arm (Figure 5H–J). Direct comparison of HT1080 huFAP GFP expression levels after transduction with either AAV2, rAAV-2E3.v6, or rAAV-2E3.v6 coupled with bispecific antibodies (KiH-2E3-MO33 or KiH-2E3-MO36 at 2.5 ng/µL) indicated a significant reduction of transduction of rAAV-2E3.v6 compared to AAV2 (mean percent of AAV-2E3.v6 GFP positive cells 0.83 ± 0.11; mean percent of AAV2 GFP positive cells 97.81 ± 0.92 *p* < 0.001). Bispecific antibodies KiH-2E3-MO33 or KiH-2E3-MO36 complexed with rAAV-2E3.v6 increased the proportions of transduced cells 13× to 57× (mean AAV-2E3.v6:KiH-2E3-MO36 GFP positive cells 57.7% ± 11.9% *p* < 0.001).

**Figure 6:**
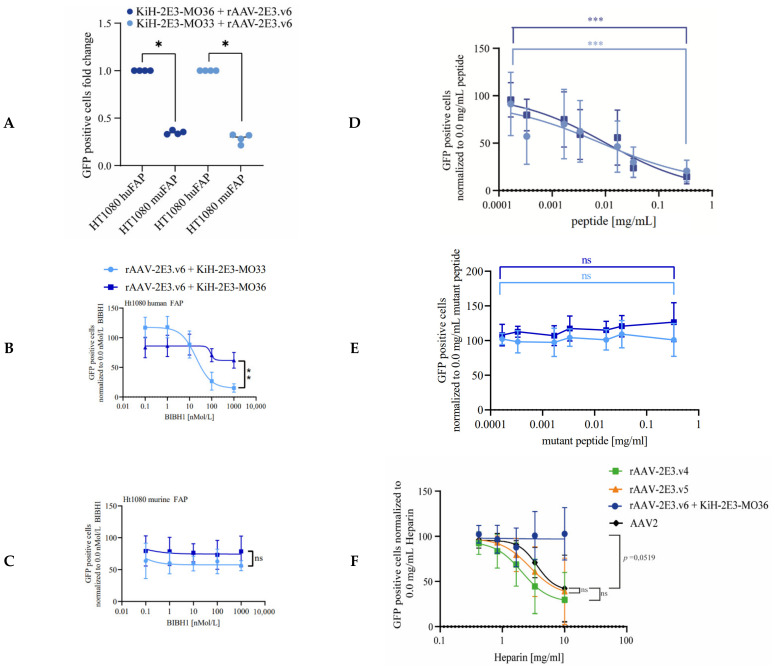
Retargeting efficiency of rAAV-2E3.v6 was affected by competitors and resists heparin binding. Cells were incubated with AAV variants and competitor agents following flow cytometry analysis of GFP expression after 3 days. Data were normalized to transduction levels without competitors. (**A**) Comparison of bispecific antibody induced rAAV-2E3.v6 transduction of HT1080 cells expressing human or murine FAP. The fold change of GFP expression was calculated. Scattered plot and mean of two independent experiments, Mann–Whitney test, * *p* < 0.05. (**B**) Specific interaction of rAAV-2E3.v6:bispecific antibodies with FAP was analyzed in competition with increasing concentrations of BIBH1 on huFAP and (**C**) muFAP expressing cells. Data show mean + SD of three independent experiments, Mann–Whitney test, ** *p* < 0.01, ns = non-significant. (**D**) Specific interaction of rAAV-2E3.v6:bispecific antibodies with 2E3 epitopes was analyzed in competition with soluble 2E3 peptide. Data show mean + SD of four independent experiments, Mann–Whitney test, *** *p* < 0.001. (**E**) Specific interaction of rAAV-2E3.v6:bispecific antibodies with 2E3 epitopes was analyzed in competition with soluble 2E3 peptide mutation. Data show mean + SD of four independent experiments, Mann–Whitney test, ns = non-significant. (**F**) Transduction of rAAV-2E3.v4, .v5, and v.6:KiH-2E3-MO36 in comparison to AAV2 under increasing concentrations of heparin-sodium in cell culture media of HT1080 huFAP cells. Data show mean + SD of four independent experiments, Mann–Whitney test, ns = non-significant.

MO36 and MO33 antibodies are described to interact with human FAP; MO36 additionally interacts with murine FAP with similar affinity, while MO33 shows reduced affinity to murine FAP [82]. Indeed, retargeting of rAAV-2E3.v6 complexed with the respective bispecific antibodies resulted in GFP positive HT1080 huFAP and muFAP expressing cells. However, a significant decrease in transduction efficiency of HT1080 muFAP cells compared to HT1080 huFAP cells was observed for both bispecific antibody variants (fold change HT1080 muFAP + rAAV-2E3.v6 + KiH-2E3-MO36: 0.35 ± 0.02; *p* < 0.05 and fold change HT1080 muFAP + rAAV-2E3.v6 + KiH-2E3-MO33: 0.28 ± 0.04; *p* < 0.05) (Figure 6A). The antibody BIBH1 is described to interact with human FAP only and was previously used as a competing antibody for FAP binding assays in cell culture [59,83]. To quantitatively assess the impact of receptor availability for this retargeting approach, HT1080 huFAP and HT1080 muFAP cells were pre-incubated with BIBH1 followed by incubation with rAAV-2E3.v6 bound by the indicated KiH antibodies. BIBH1 was able to reduce transduction of HT1080 huFAP cells in a concentration dependent manner but had no effect on transduction of muFAP expressing cells (Figure 6B,C), as expected, since it failed to interact with murine FAP. Furthermore, BIBH1 showed stronger competition with KIH MO33 variants, possibly not only by reducing FAP receptor availability but also by overlapping FAP epitopes recognized by the respective antibodies. In conclusion, we generated novel rAAV2 capsid modifications that were bound by bispecific antibodies for successful retargeting to human FAP receptor positive cells.

Transduction dependence upon 2E3 epitope binding was analyzed by competing the rAAV-2E3.v6 antigen binding with soluble 2E3 peptides in vitro. For this purpose, bispecific antibodies and rAAV-2E3.v6 particles were pre-incubated in the presence of increasing concentrations of 2E3 and control peptides followed by incubation of HT1080 huFAP cells with the formed complexes. Analysis of GFP expression by flow cytometry revealed a significant reduction of GFP expression with increasing concentrations of soluble 2E3 peptide (rAAV-2E3.v6 + KiH-2E3-MO33 + 0.33 mg/mL 2E3 peptide: 20.42% ± 11.62% GFP positive cells; *p* < 0.001 and rAAV-2E3.v6 + KiH-2E3-MO36 + 0.33 mg/mL 2E3 peptide:14.21% ± 7.95% GFP positive cells; *p* < 0.001) (Figure 6D). This effect could not be seen when a mutated, non-binding 2E3 epitope was used (Figure 6E). These results indicated that the KiH-2E3-MO33 and the KiH-2E3-MO36 antibodies were sensitive to amino acid changes of the 2E3 epitope and that capsid binding via the integrated 2E3 peptide sequences was essential for productive rAAV-2E3 target cell transduction.

Finally, we demonstrated that soluble heparin did not affect the transduction of rAAV-2E3.v6:KiH-2E3-MO36, while increasing concentrations of heparin inhibited the cellular transduction of AAV2, rAAV-2E3.v4, and rAAV-2E3.v5 (Figure 6F). Therefore, we concluded that an insertion independent of the length of the epitope (rAAV-2E3.v4 and rAAV-2E3.v5) did not affect heparin or HSPG binding of viral capsids, while substitution of the RXXR motif in VP proteins (rAAV-2E3.v6) dramatically impeded the HSPG interaction. These in vitro data support our initial findings showing reduced heparin interaction of rAAV-2E3.v6 but similar binding of rAAV-2E3.v4 and rAAV-2E3.v5 with heparin columns compared to AAV2.

In summary, we showed that our bispecific antibody:rAAV-2E3.v6 targeting approach was receptor- and epitope-specific, and transduction efficiency depended on the antibody–epitope interaction.

### 2.5. Substitution of the Receptor Binding Antibody Fragment Enables Targeting of PD-L1 Expressing Cells

**Figure 7:**
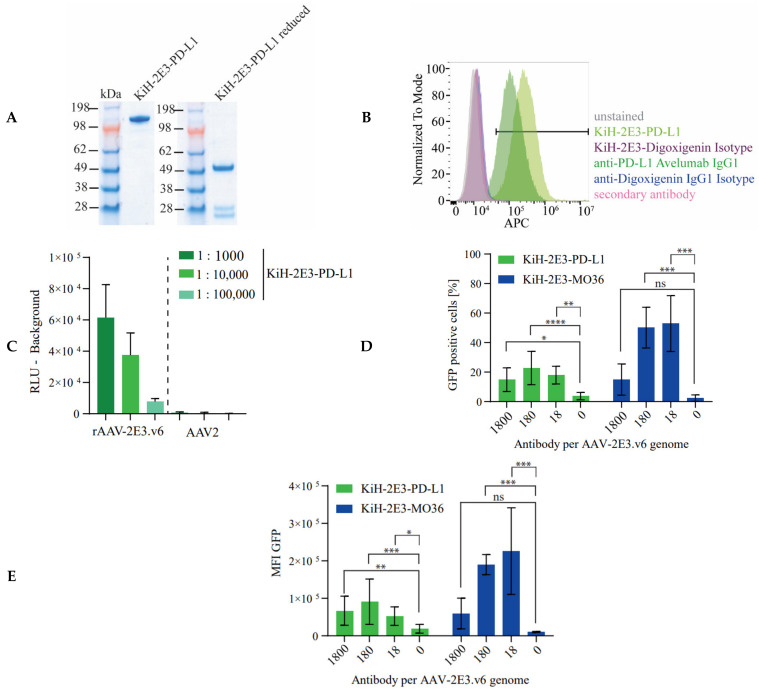
Bispecific antibody modification enabled modular targeting of PD-L1 expressing cells with rAAV-2E3.v6. (**A**) A new bispecific antibody KiH-2E3-PD-L1 was produced and purified. The size difference of kappa and the shorter lambda light chains were visible under reducing conditions. (**B**) Flow cytometry staining of HT1080 cells proved expression of PD-L1 using avelumab, KiH-2E3-PD-L1, and isotype controls. (**C**) KiH-2E3-PD-L1 antibody binding of immobilized rAAV-2E3.v6 and AAV2 capsids. Data show mean + SD of two independent experiments. (**D**) Flow cytometry analysis of GFP expressing HT1080 huFAP cells after re-targeting of rAAV-2E3.v6 by KiH-2E3-PD-L1 in comparison to KiH-2E3-MO36. Plot of GFP positive cell proportoins and (**E**) MFI levels. Data show mean ± SD of three independent experiments; one-way ANOVA, uncorrected Dunn’s test, * *p* < 0.05; ** *p* < 0.01; *** *p* < 0.001, **** *p* < 0.0001.

After successfully targeting FAP, we decided to redirect rAAV-2E3.v6 to an unrelated cell surface antigen on the same cell line using a simple “Fab-arm exchange” in our bispecific antibody to change target specificity. We chose PD-L1 as cell surface marker, which is associated with many cancers [60,62,89]. Antibody sequences recognizing PD-L1 (avelumab) are publicly available, and the interaction of avelumab and PD-L1 was extensively validated in various (pre-)clinical studies [90]. In addition, internalization of PD-L1 upon avelumab binding was also described earlier [84]. Finally, HT1080 cells were reported to express PD-L1 [91,92]. We successfully produced a bispecific antibody composed of the knob-anti-2E3 arm hybridized with the hole-anti-PD-L1 arm (Figure 7A). We confirmed PD-L1 expression by HT1080 huFAP cells using flow cytometry comparing the KiH-2E3-PD-L1 antibody with avelumab and corresponding isotype controls (avelumab: 92.8%; KiH-2E3-PD-L1: 99.0% APC positive cells) (Figure 7B). The bispecific antibody bound the 2E3 epitope of immobilized rAAV-2E3.v6, but no interaction with the parental AAV2 capsids was observed (Figure 7C). The anti-VP antibody A20 was used as a control to ensure equivalent immobilization of rAAV-2E3.v6 and AAV2 capsids (Figure S4). Finally, we successfully retargeted rAAV-2E3-v.6 via KiH-2E3-PD-L1 binding to HT1080 huFAP cells, resulting in a significant increase in GFP positive cells in comparison to control groups (mean GFP positive cells 22.73% ± 11.28%; *p* < 0.0001). Equal amounts of KiH-2E3-MO36 per rAAV-2E3.vg genome resulted in higher amounts of GFP positive cells in comparison to control groups (mean GFP positive cells 50.19% ± 13.78%; *p* < 0.001) (Figure 7D), and measurement of MFI confirmed a higher efficiency of FAP related targeting (Figure 7E). However, the targeting efficiency of KiH-2E3-PD-L1 fell within the range of KiH-2E3-MO33 and -MO36 transduced GFP positive cells.

In summary, we developed a successful retargeting approach based on a new AAV2 capsid modification. By modular combination with different bispecific antibodies, we could selectively target different cell surface receptors, leading to specific transduction of these cells.

## 3. Discussion

This study was aimed at the development of a modular platform for retargeting of AAVs. For this purpose, we designed and produced novel, detargeted rAAV2 variants, which lost their broad tropism by insertion of a short peptide epitope into capsid regions crucial for cellular transduction. To achieve this, PCSK9-derived 2E3 epitopes were inserted into five different AAV2 capsid sites. Four out of five rAAV-2E3 capsid variants could be produced with detectable epitope insertion in all VP proteins (Figure 2C). The capsid regions between 491 and 514 (rAAV-2E3.v2 and rAAV-2E3.v3) were chosen for an insertion of TPPRYRAD framed with GS-linkers to interfere with the highly conserved binding motif NGR (511–513) responsible for integrin α5β1 binding and internalization [16]. rAAV-2E3.v3 showed a low packaging capacity and was excluded from further experiments. We assumed that a capsid substitution of four amino acids (510 to 514) with 16 amino acids within this specific site interfered with efficient capsid assembly. A nearby substitution of nine amino acids (491–501) with the 2E3 epitope was well tolerated (rAAV-2E3.v2). As desired, the capsid modification had a significant impact on viral transduction efficiency, as rAAV-2E3.v2 showed no transduction of any of the tested cell lines, demonstrating efficient detargeting. However, this effect could not be reversed by our bispecific antibody targeting approach, although we could confirm bispecific antibody binding to the v2 variant. The 2E3 substitution might interfere with integrin α5β1 interaction, which could be still important for our retargeting mechanism. Additionally, this modification could influence internalization, trafficking, and unpackaging of internalized capsids and therefore could hinder gene expression. Impaired heparin interaction of rAAV-2E3.v2 compared to AAV2 could not be demonstrated in our heparin column binding assay and therefore was very likely not the reason for restricted transduction (Figure 3C). We concluded that the capsid area 491 to 514 was sensitive to substitutions, and produced rAAV-2E3 variants did not meet our criteria of both detargeting and receptor-specific retargeting [16]. 

As previously described, the position surrounding 587 is located within the variable surface region of loop eight of the AAV2 capsid and tolerates capsid modifications [11,42,45,46]. Our results support these findings, as capsid modifications independent of length, GS-linker, insertion, or substitution (rAAV-2E3.v4, .v5, and .v6) could be produced with yields comparable to the parental AAV2. Production yield of viral genomes and viral capsids showed a strong correlation, although absolute numbers were not comparable. According to our EM data and AAV production protocol, we produced rAAVs with high packaging capacity and low impurities [93].

HEK 293, B16-F10, FL8-3B, and CT26-CL25 cells were most efficiently transduced by AAV2. The transduction efficiency decreased between rAAV-2E3.v5 to rAAV-2E3.v4 regarding the cell lines and the MFI (Figure 3A,B). The data obtained from rAAV-2E3.v4 and rAAV-2E3.v5 indicated that longer insertions at position 588 resulted in a more pronounced decrease in cell transduction efficiency, as the insertion of rAAV-2E3.v4 was eight amino acids longer than in rAAV-2E3.v5. rAAV-2E3.v6 did not show transduction of cells at any VG/cell. Our modification, a substitution between 581 and 589, dramatically inhibited natural transduction, which is in line previous findings [42]. This impaired transduction pattern could be related to significantly reduced interaction with heparin compared to AAV2. Basic amino acids R484, R487, R585, R588, and K532 of the AAV2 capsid interacted with negatively charged sulfate and carboxyl groups [13,85]. rAAV-2E3.v6 lost R585 and R588 due to substitution of an epitope with neutral charge, while the motif remained intact for rAAV-2E3.v2, .v4, and .v5 that did not show impaired heparin interaction (Figure 3C and Figure 6F).

Four knob-into-hole bispecific antibodies were developed that all shared the same arm binding the 2E3 epitope (“knob”) but differed in their retargeting arm (“hole”) binding of different receptors FAP (MO33 and MO36), PD-L1 (avelumab), or the molecule digoxigenin as isotype control. This format allowed for fast and flexible production of bispecific antibodies [87] by combining the permanent “knob” 2E3 specific Fab fragment with various “hole” variants. We demonstrated that our bispecific antibodies specifically bound capsid inserted 2E3 (Figure 4D and Figure 7B) as well as assigned target receptors FAP or PD-L1 with equal levels as compared to parental, monospecific antibody controls (Figure 4B,C and Figure 7B). Purified bispecific antibodies simultaneously bound 2E3 peptides and recombinant FAP in a bridging assay without any loss of specificity compared to the monospecific parental antibodies. Furthermore, the internalization of bispecific antibodies could be demonstrated on HT1080 huFAP expressing cells as described earlier. FAP internalized upon antibody binding within 3 h [81], which confirmed previous findings of antibody mediated endocytosis upon FAP binding [72,94]. We produced bispecific antibodies that showed specific epitope binding comparable to parental monospecific antibodies and allowed for flexible engagement of receptors.

Our targeting approach was established using HT1080 cells stably expressing FAP [82]. KiH-2E3-FAP antibodies (MO33 or MO36) were titrated against different VG/cell of rAAV-2E3 variants, and GFP expression was measured three days later by flow cytometry. Incubation of AAV2 with any of these bispecific antibodies did not interfere with viral transduction, indicating no unspecific interaction of the bispecific antibodies with the viral capsid or the cells (Figure 5G). As rAAV-2E3.v4 and rAAV-2E3.v5 showed a high background of GFP positive cells, a bispecific antibody mediated targeting effect could not be observed. However, high amounts of bispecific antibodies binding viral capsids showed a trend to reduced transduction frequencies, which was possibly due to generation of large antibody–rAAV-2E3 complexes which may have inhibited internalization or promoted degradation by the proteasome after internalization. rAAV-2E3.v6 on its own did not induce GFP expression in cells, as expected, but increasing amounts of GFP positive cells and consequently increasing MFIs were directly linked with the ratio of KiH-2E3-MO33 or KiH-2E3-MO36 molecules per vial genome (Figure 5A,F–H). As this effect was not observed after incubation with bispecific isotype control antibodies or in HT1080 cells lacking FAP expression (Figure 5H–K), we concluded that this transduction was the result of specific antibody mediated FAP targeting. 

The optimal ratio of bispecific antibodies per rAAV-2E3.v6 genome can be visualized in a dose-response curve. Insufficient bispecific antibody:capsid (<120:1) and high antibody:capsid ratios (>1200:1) decreased transduction, presumably due to inadequate binding events or excessive amounts of unbound bispecific antibodies that blocked FAP receptors. Efficient retargeting was achieved between 180 and 600 antibody molecules per viral genome, notably, rAAV-2E3.v6:KiH-2E3-MO36 achieved higher frequencies of GFP positive cells and higher MFIs compared to KiH-2E3-MO33 mediated retargeting. These differences were possibly due to distinct FAP epitopes and affinities [82]. Based on the bispecific antibody excess required for efficient retargeting and 2E3-antibody binding curves (Figure 4E–G), we concluded that the 2E3 epitope was not bound with high affinity, and targeting of cells most likely required additional adapter molecules to facilitate internalization.

Both MO33 and MO36 bispecific antibodies bound human and murine FAP [82], but retargeting efficiency to murine FAP was somewhat reduced for both antibodies. This difference was possibly related to lower expression levels of murine FAP compared to huFAP or a reduced affinity to muFAP, as described for MO33 [82]. We could prove FAP specific targeting within a competition experiment using the BIBH1 antibody described for human but not murine FAP [59,83]. BIBH1 competition strongly inhibited KiH-2E3-MO33 mediated huFAP targeting and had a mild effect on KiH-2E3-MO36 FAP targeting, while targeting of murine FAP was unaffected by any BIBH1 concentration (Figure 6B,C). Inhibition of MO33 induced retargeting of cells could have been due to overlapping epitopes and reduced receptor availability [82]. Therefore, we suggest that both affinity and epitope of the bispecific antibody affect targeting efficiency. The targeting effect was highly specific and dependent on the correct epitope interaction of the antibody, as proven by KiH-2E3-FAP competition with soluble 2E3 epitopes, resulting in decreased amounts GFP positive cells, while soluble mutated 2E3 peptides showed no effect on transduction efficiencies (Figure 6D,E).

Having established a bispecific antibody targeting system based on rAAV-2E3.v6, we demonstrated that this system can be adapted to target other membrane anchored proteins. By using an appropriately designed bispecific antibody (KiH-2E3-PD-L1), we showed rAAV-2E3.v6 capsid binding as well as staining of HT1080 PD-L1 positive cells. Successful PD-L1 targeting was achieved at similar ratios of antibody per viral genome, as observed for FAP targeting by KiH-2E3-MO36. Lower rates of GFP positive cells and consequently MFIs were observed in a direct comparison with KiH-2E3-MO36 but not in comparison to KiH-2E3-MO33 targeted cells. Differences in GFP positive cells and MFI possibly resulted from different expression levels of the native expressed PD-L1 compared to stably expressed FAP or different internalization rates. Furthermore, we cannot exclude the possibility that the interactions with co-receptors such as integrin α5β1 are still important for internalization. In theory, the suitability of other potential cell surface antigens for retargeting depends on internalization rates, antigen density, epitope, and affinity of the selected retargeting antibody. Our observations with MO33 and MO36 on human and murine FAP support this hypothesis. AAV serotypes share a common capsid structure, a high degree of identical as well as homologous regions, and variable surface loops that are described to be equally suited for capsid engineering [95,96]. Therefore, we assume that our 2E3 epitope substitution and modular retargeting approach is applicable for various AAV serotypes.

This targeting approach demonstrated highly specific receptor targeting based on initial restriction of the viral tropism. Our targeting approach showed reduced GFP expression levels (as measured by MFIs) compared to AAV2, which, however, was mediated by abundant HSPG binding. Presumably, defined cell surface targets such as FAP and PD-L1 used here are present at lower levels compared to HSPG, and different HSPG-modified proteins have heterogenous endocytosis rates, further contributing to transduction differences compared to AAV2. Moreover, one can speculate that viral trafficking and unpackaging could be altered by our capsid modification and targeting approach, resulting in reduced expression levels.

In summary, we developed a new rAAV-2E3.v6 capsid variant based on AAV2 capsid modification by insertion of the 2E3 epitope derived from PCSK9. The novel capsid variant showed strongly reduced binding to heparin, resulting in strongly altered tropism compared to the parental AAV2. Transduction could be restored by pre-incubation with a bispecific antibody binding both the inserted epitope and a selected cell surface receptor. This retargeting mechanism could enable the specific targeting of different cells in mixed cultures or tissues by exchange of the retargeting antibody with the same reusable viral variant and provides a valuable tool for investigating various biological processes as well as a basis for novel gene therapy approaches.

## 4. Materials and Methods

### 4.1. Expression and Capsid Constructs

The expression plasmid pFBsc-CMV-eGFP consisted of a CMV promoter followed by a Kozak sequence, a GFP reporter gene, and a SV40 polyA signal. The expression cassette was flanked by AAV2 ITRs, from which one was lacking the terminal resolution site. Consequently, the rAAVs contained a self-complementary viral genome. The AAV2 cap gene was modified by inserting variations of the PCKS9 epitope ITPPRYRADE at different capsid sites each (Table 1). Sequences were synthesized by GeneArt (Thermo Fisher Scientific Waltham, MA, USA) and cloned into a plasmid co-encoding the AAV2 rep gene based on standard AAV helper constructs. Knob-into-hole antibodies were designed according to [87], anti-PCSK9 (2E3) antibody sequences were described by [50], and anti-FAP (MO33 and MO36) antibody sequences were derived from [82]. The PD-L1 antibody (Avelumab SB0010718C) sequence was derived from DrugBank. The anti-digoxigenin antibody sequence was described previously [97].

### 4.2. Production of huFAP and muFAP Positive Cell Lines

HT1080 cells were derived from ATCC and cultured in RPMI 1640 media (Thermo Fisher Scientific Waltham, MA, USA) supplemented with 10% FCS (Thermo Fisher Scientific Waltham, MA, USA) and 1× MEM Non-Essential Amino Acids Solution (100X) (Thermo Fisher Scientific Waltham, MA, USA). Cells were transfected with Lipofectamine™ 2000 Transfection Reagent (Thermo Fisher Scientific Waltham, MA, USA) and expression constructs of human or murine FAP and selected by 300 µg/mL Geneticin™ Selective Antibiotic (Thermo Fisher Scientific Waltham, MA, USA) as described previously [64].

### 4.3. Antibody Production

HEK293-E6 suspension cells were licensed from the National Research Council (NRC) of Canada and described previously [98]. Cells were cultured in FreeStyle™ F17 Expression Medium (Thermo Fisher Scientific Waltham, MA, USA) supplemented with 1× GlutaMAX™ Supplement, 0.1% Pluronic™ F-68 Non-ionic Surfactant, and 0.25 µg/mL Geneticin™ Selective antibiotic (all purchased from Thermo Fisher Scientific Waltham, MA, USA) at 37 °C and 5% CO_2_ at 120 rpm. A total of 24 h before transient transfection, the cell culture media was changed to antibiotic free media. Transient transfection was performed with 293fectin™ Transfection Reagent (Thermo Fisher Scientific Waltham, MA, USA) with 1.0 µg DNA and 1.0 µL 293fectin™ per mL cell culture volume. Production was performed separately for knob (HC and LC) and hole (HC and LC) constructs. Then, 24 h after transfection, cells were fed with 0.3 mL 40% Tryptone N1 per mL cell culture volume (Sigma-Aldrich, St. Louis, MO, USA). After six days of production, the supernatant was harvested, and IgGs were purified via ÄKT Avant™ 25 (Cytiva, Marlborough, MA, USA) and HiTrap™ MabSelect™ SuRe 5 mL column (Cytiva, Marlborough, MA, USA). Fab-arm exchange was performed to produce knob-into-hole bispecific antibodies according to previous publication [87]. In short, equimolar amounts of knob and hole purified antibodies were mixed in a final concentration of 25 nM 2-MEA (Sigma-Aldrich, St. Louis, MO, USA) in PBS for 5 min at RT under rotation and following 5 h at 25 °C without agitation. The buffer was exchanged via Amicon^®^ Ultra-30 Centrifugal Filter Units (Merck Merck Millipore, Burlington, MA, USA) to the desired storage buffer (PBS). Bispecific antibodies were stored at 4 °C overnight for re-oxidation of disulfide bonds. In order to remove unpaired or hole-hole paired antibodies, a cation-exchange chromatography using a HiTrap SP FF 1 mL (Cytiva, Marlborough, MA, USA) was performed in 50 mM sodium acetate running buffer (pH 5.0) and 50 mM sodium acetate 1 M NaCl elution buffer (pH 5.0) via ÄKTA Avant™ 25 in order to separate proteins by their isoelectric point. Finally, size-exclusion chromatography was performed to remove any protein aggregations by using a HiLoad^®^ 26/600 Superdex^®^ 200 pg column (Cytiva, Marlborough, MA, USA) in PBS. The desired antibody fractions were pooled and analyzed by SDS PAGE under reducing and non-reducing conditions. Staining of proteins was performed with Expedeon InstantBlue™ Stain Protein Stain (Thermo Fisher Scientific, Waltham, MA, USA). Purified antibodies were tested endotoxin free using the PTS20 LAL Test Cartridges and Endosafe -PTS™ system (Charles River, Wilmington, MA, USA) according to the manufacturer’s manual, and only antibodies with a endotoxin concentration >5.0 EU/mL were used for cell culture experiments.

### 4.4. Octet Analysis of Bispecific Antibodies

To confirm antibody bispecificity, a binding assay via Octet HTX (PALL^®^ Laboratory) was performed using a TW384 Microplate (foreBio) containing 10.0 µg/mL of each protein or peptide. A biotin-2E3 epitope (Biotin-ITPPRYRADE) (EMC Microcollections, Tübingen, Germany) was bound to a streptavidin biosensor (ForteBio, Fremont, CA, USA). The bound bispecific antibody enabled further binding of soluble recombinant human FAP (Enzo Lifesciences, Farmingdale, NY, USA) and BSA-conjugated digoxin (Biozol, Eching, Germany).

### 4.5. Production and Analysis of Recombinant AAV Vectors

The recombinant AAV vectors were produced by calcium phosphate transfection of human embryonic kidney cells (HEK 293-H cells, Thermo Fisher Scientific Waltham, MA, USA) using a three-plasmid based production protocol (AAV helper free system; Agilent Technologies, Santa Clara, CA, USA) and purified as described [99] Vector yield was analyzed by ddPCR, and viral titers were determined by ELISA (Progen, Heidelberg, Germany) according to the manufacturer’s manual.

### 4.6. ddPCR

ddPCR was performed to measure viral genomes. Viral DNA was purified using the ViralXpress™ Nucleic Acid Extraction Kit (Merck Merck Millipore, Burlington, MA, USA) according to manufacturer’s instructions. A ddPCR was performed with samples after a serial dilution in 1:10 steps in DEPC-treated water and mixed with 1× ddPCR™ Supermix for Probes (Bio-Rad, Hercules, CA, USA) and 1× Primer-Probe-Mix for detection of CMV. A final concentration of 0.9 µM of each primer was used (forward primer CCAAGTACGCCCCCTATTGAC, reverse primer: CTGCCAAGTAGGAAAGTCCCATAAG) in total of 0.25 µM including the probe (FAM-CCGCCTGGCATTATG-MGB). The AutoDG™ (Bio-Rad, Hercules, CA, USA) was used to created droplets in a 96-well format following a PCR reaction. The droplets were measured by the QX200™ Droplet Reader (Bio-Rad, Hercules, CA, USA)

### 4.7. Meso-Scale Discovery^®^-Electrochemiluminescence ELISA Assay

Interaction of anti-2E3 antibody and AAV-2E3 epitope was analyzed using the meso-scale discovery platform. The viral variants were diluted in 1× PBS, and 30.0 µL were immobilized on a MSD^®^ standard plate with a concentration of 5.0 × 10^8^ capsids/well at 4 °C overnight. The plate was washed three times with wash buffer (0.05% Tween20 in PBS) and then blocked in 3.0% Blocker A (MSD^®^ Rockville, MD, USA) in PBS for 1 h at RT. The primary antibody was diluted in 1% Blocker A in PBS and incubated for 1 h at RT. Secondary antibodies (MSD^®^ Rockville, MD, USA) were diluted in 1% Blocker A in PBS 1:500 (anti Mouse Antibody Goat SULFO-TAG) or 1:1000 (anti Human Antibody Goat SULFO-TAG), respectively, for 1 h at RT. The 2× read buffer in H_2_O was added to measure the electrochemical signals using the MSD^®^ Sector imager 6000 (MSD^®^ Rockville, MD, USA).

### 4.8. Western Blot

SDS-PAGE was performed with rAAV particles (1 × 10^10^ VG) under reducing conditions and blottet to a membrane using the iBlot Dry Blotting System (Invitrogen™, Carlsbad, CA, USA) and iBlot™ Trans-fer Stack (Invitrogen™, Carlsbad, CA, USA). The membrane was blocked with 5.0% milk in PBS-T (0.1% Tween in PBS) for 1 h at RT following first antibody incubation with primary antibody B1 monoclonal mouse anti VP1, VP2, VP3 (Progen, Heidelberg, Germany) 1:250 and anti-2E3 IgG1 human (own production) 1:250 in 1.0% milk PBS-T overnight at 4 °C. Secondary antibodies anti-human IgG Fc HRP or goat anti-mouse IgG1 (H+L) HRP (Thermo Fisher Scientific Waltham, MA, USA) were used 1:1000 in 1.0% milk PBS-T for 1 h at RT. The SuperSignal™ West Pico PLUS Chemiluminescent Substrate (Thermo Fisher Scientific Waltham, MA, USA) was used to detect chemiluminescent signals by the Image Quant LAS 4000 (Cytiva, Marlborough, MA, USA).

### 4.9. In Vitro rAAV Transduction Assays

For AAV particle transduction experiments, cells were plated in ViewPlate-96 Black (PerkinElmer^®^, Waltham, MA, USA) at cell type specific densities to ensure no overgrowth. The cells were infected in 1/3 dilutions of 150,000 VG/cell to 5000 VG/cell. Successful transduction was analyzed via IncuCyte^®^ S3 Live-Cell Analysis System (Sartorius, Göttingen, Germany) and flow cytometry after three days. Cell lines 4T1, B16-F10, bEND.3, CT26-CL25, NIH3T3, Renca, Tramp C2, and FL8-3B were purchased from ATCC (Manassas, VA, USA). MC-38 cells were purchased from NIH/NCI (Bethesda, MD, USA).

### 4.10. Retargeting of AAV by Bispecific Antibodies

In total, 5000 cells per well were plated into a ViewPlate-96 Black (PerkinElmer^®^, Waltham, MA, USA) 24 h before transduction. The bispecific antibody was diluted in a serial dilution starting with 15.0 ng/µL equal to 1.0 nMol/L to 1.5 pg/µL in 1.0% FCS in PBS. For transduction of 10,000 cells, 15 µL of rAAV particles diluted in PBS 1.0% FCS of indicated VG/cell and 15 µL of bispecific antibody of indicated dilutions were pre-incubated for 1 h at 37 °C in a V-bottom 96-well plate. The plate used for pre-incubation was blocked for at least 1 h at 37 °C with pure FCS. Then, 20.0 µL of pre-incubated rAAV and bispecific antibody was added to 10,000 cells following incubation in a wet chamber for three days.

Competition assays were performed using BIBH1 antibody (Boehringer Ingelheim Pharma KG, Biberach Riß, Germany), soluble epitopes 2E3 peptide (ITPPRYRADK-Biotin-Aca), 2E3 mutant epitope (ITPPRARYDK-Biotin-Aca) (EMC Microcollections, Tübingen, Germany), or heparin-sodium (Sigma-Aldrich, St. Louis, MO, USA) diluted in cell culture media. Competitors (antibodies and peptides) were diluted in 1.0% FCS in PBS and 15 µL pre-incubated with bispecific antibodies and rAAVs in equal volumes. In total, 30.0 µL of the pre-incubation was added to 10,000 cells following incubation in a wet chamber for three days.

### 4.11. Flow Cytometry

Expression of surface markers was analyzed by antibody staining and flow cytometry analysis. Cells were detached from the culture plate using Accutase (Invitrogen™, Carlsbad, CA, USA) and collected in a round bottom 96-well plate in FACS buffer containing 1× PBS and 1.0% FBS (Thermo Fisher Scientific Waltham, MA, USA). Fc-receptors were blocked with 1.0% human FcR Blocking Reagent (Miltenyi Biotec, Bergisch Gladbach, Germany) in FACS buffer. Cells were incubated with 0.5 µg primary antibody for 30 min on ice, washed with FACS buffer, and incubated with a 0.5 µg secondary antibody APC anti-human IgG Fc (BioLegend^®^, San Diego, CA, USA) for 30 min on ice. Cells were washed and analyzed by BD FACSCanto™ II Flow Cytometer and FlowJo™ V10 software.

Cells transduced with a GFP transgene were detached from the culture plate with Accutase (Invitrogen™, Carlsbad, CA, USA) and transferred into a round bottom 96-well plate in FACS buffer. GFP expression was measured using the iQue^®^ Screener PLUS (Sartorius, Göttingen, Germany).

### 4.12. Transmission Electronmicroscopy

The 10.0 µL virus suspensions were incubated on a pre-cleaned grid for 1 min, and the residual fluid was removed by filter paper. Negative staining of rAAVs was performed with 10 µL of 2.0% phosphotungstic acid (Sigma-Aldrich, St. Louis, MO, USA) in ddH_2_O (pH 7.0) for 1 min incubation time. Finally, the samples were analyzed using a transmission electron microscope EM 912 (Zeiss SMT, Oberkochen, Germany).

### 4.13. Heparin-Binding Assay

Interaction of novel rAAV2 particles with Heparin-Agarose (Sigma-Aldrich, St. Louis, Missouri, USA-Aldrich, St. Louis, MO, USA) was analyzed according to [85]. In short, Micro Bio-Spin^®^ Columns (Bio-Rad, Hercules, CA, USA) were blocked overnight in 100% FCS and packaged with 500 µL Heparin-Agarose each. The column was washed with AAV buffer (1× PBS, 1 mM MgCl_2_, 2.5 mM KCl, 10% glycerol, 0.001% Pluronic, pH 7.4) trice. Viral particles were diluted in AAV buffer to an amount of 5 × 10^10^ in total 600 µL volume. Then, 100 µL of the load volume were stored for later analysis, and 500 µL were loaded to prepared columns. The column was washed five times with 500 µL AAV buffer following elution with 500 µL AAV buffer plus 2 M NaCl. Viral DNA was extracted using the ViralXpress™ Nucleic Acid Extraction Kit (Merck Millipore, Burlington, MA, USA) according to manufacturer’s instructions. Purified DNA was analyzed by ddPCR.

### 4.14. Antibody Internalization Assay

Cells were detached from culture plates by Accutase (Invitrogen™, Carlsbad, CA, USA) treatment and were seeded at a density of 10,000 per well 4 h before assay start in a ViewPlate-96 Black (PerkinElmer^®^, Waltham, MA, USA) in 50 µL appropriate cell culture media per well. IncuCyte^®^ FabFluor pH Red Antibody Labeling reagent (Sartorius) was rehydrated in ddH2O to a final concentration of 0.5 mg/mL. Antibodies were used at a final concentration of 4 µg/mL and mixed in a 1:3 molar ratio with FabFluor pH Red Antibody Labeling reagent for 15 min at RT in appropriate cell culture media. Cell culture media was removed from cells and replaced with labeled antibody media. Cells were analyzed by in an IncuCyte^®^ live-cell analysis system with a 10× objective in channel “phase” and “red” all 15 min within 5 h.

### 4.15. Statistical Analysis

Statistical calculations were performed using GraphPad Prism software version 9.0 (GraphPad Software, Inc., San Diego, CA, USA). Data were presented as mean ± standard deviation or mean ± standard error of mean (Figure 3C). Figure 3C shows analysis by one-way ANOVA and uncorrected Fisher’s LSD. Figure 5D–F,H–J,L and Figure 7D,E show analysis by one-way ANOVA and uncorrected Dunn’s test. Figure 6A–F show Mann–Whitney test. *p*-values are denoted as follows: * *p* < 0.05, ** *p* < 0.01, *** *p* < 0.001, and **** *p* > 0.0001; ns: non-significant *p* > 0.05.

## Figures and Tables

**Table 1:** Design of novel AAV2 based viral capsids via peptide insertion.

Amino Acid Position	Original Sequence/*Insertion*	Novel rAAV
Sub491-501-GS4	**VSKT** *GGGS TPPRYRAD GGGS* **SWTG**	rAAV-2E3.v2
Sub510-514-GS4	**YHL** *GGGS TPPRYRAD GGGS* **DSL**	rAAV-2E3.v3
Ins588-GS4	**TNLQRGNR** *GGGS TPPRYRAD GGGS* **QAA**	rAAV-2E3.v4
Ins588	**TNLQRGNR** *GTPPRYRAD* **QAA**	rAAV-2E3.v5
Sub581-589-GS4	**T** *GGGS ITPPRYRAD GGGS* **QAA**	rAAV-2E3.v6

## Data Availability

Not applicable.

## Supplementary Materials

The following are available online at https://www.mdpi.com/article/10.3390/ijms22158355/s1.

## Abbreviations

2-MEA	2-mercaptoethylamine-HCl
AAP	assembly activating protein
(r)AAV2	(recombinant) adeno-associated virus type 2
CH	heavy chain
ddPCR	droplet digital polymerase chain reaction
Fab	fragment antigen binding
FAP	fibroblast activated protein
Fc	fragment crystallizable region
HSPG	heparan sulfate-proteoglycan
Hu	human
LC	light chain
MSD^®^-ECL	meso scale discovery electrochemiluminescence
mu	murine
NaCl	sodium chloride
ns	Non-significant
PD-L1	programmed cell death 1 ligand 1
RT	room temperature
scFv	single chain variable fragment
VG	viral genomes
VP	viral protein
