# Supplementary material for: Development of a Bispecific Antibody-Based Platform for Retargeting of Capsid Modified AAV Vectors
Source: Int J Mol Sci. 2021 Aug 3;22(15):8355. doi: 10.3390/ijms22158355 (PMC8347852; doi:10.3390/ijms22158355)
Supplement: Supplementary file 1 [file ijms-22-08355-s001.zip › ijms-1273905-supplementary.pdf]

International Journal of *Molecular Sciences*

MDPI

Supplementary Material

Development of a bispecific antibody-based platform for retargeting of capsid modified AAV vectors

Juliane Kuklik [1], Stefan Michelfelder [2], Felix Schiele [3], Sebastian Kreuz [2,4], Thorsten Lamla [5], Philipp Müller [4,†] and John E. Park [1,*,†]

[1] Division of Cancer Immunology and Immune Modulation, Boehringer Ingelheim Pharma GmbH & Co. KG, 88387 Biberach an der Riss, Germany; juliane.kuklik@boehringer-ingelheim.com
[2] Division of Research Beyond Borders, Boehringer Ingelheim Pharma GmbH & Co. KG, 88387 Biberach an der Riss, Germany; stefan.michelfelder@boehringer-ingelheim.com (S.M.); sebastian.kreuz@boehringer-ingelheim.com (S.K.)
[3] Division of Biotherapeutics Discovery, Boehringer Ingelheim Pharma GmbH & Co. KG, 88387 Biberach an der Riss, Germany; felix.schiele@boehringer-ingelheim.com
[4] Boehringer Ingelheim Venture Fund GmbH, 55216 Ingelheim am Rhein, Germany; philipp_3.mueller@boehringer-ingelheim.com
[5] Division of Drug Discovery Sciences Biberach, Boehringer Ingelheim Pharma GmbH & Co. KG, 88387 Biberach an der Riss, Germany; thorsten.lamla@boehringer-ingelheim.com
* Correspondence: john.park@boehringer-ingelheim.com
† These authors contributed equally to this work.

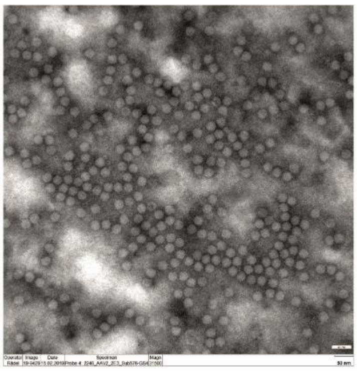

Figure 1. Transmission electron microscopy of negative stained rAAV-2E3.v6 viral particles, uncropped image. Scalebar 50 µm.

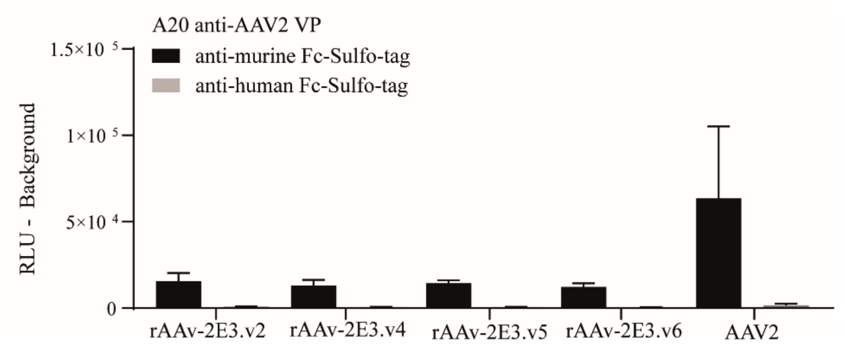

Figure 2. MSD ELISA control staining of immobilized rAAV-2E3.v2, -v4, -v5, -v6 and AAV2 following incubation with A20 anti-VP antibody and anti-murine Fc-Sulfo-tag secondary antibody. Loading control of Figure 2E. The secondary anti-human Fc-Sulfo-tag was used as isotype control. Data represent mean + SD of three independent experiments.

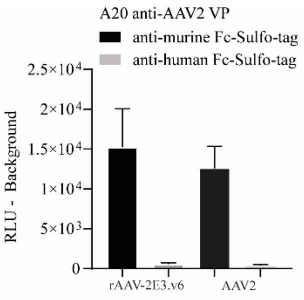

Figure 3. MSD ELISA control staining of immobilized rAAV-2E3. v6 and AAV2 following incubation with A20 anti-VP antibody and anti-murine Fc-Sulfo-tag secondary antibody. Loading control of Figure 4D The secondary anti-human Fc-Sulfo-tag was used as isotype control. Data show mean ± SD of three independent experiments.

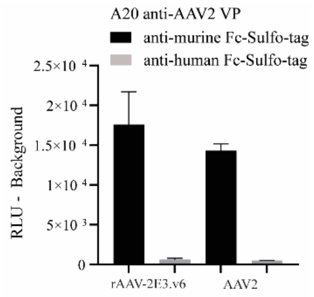

Figure 4. MSD ELISA control staining immobilized rAAV-2E3. v6 and AAV2 following incubation with A20 anti-VP antibody and anti-murine Fc-Sulfo-tag secondary antibody, loading control of Figure 7C. The secondary anti-human Fc-Sulfo-tag was used as isotype control. Data show mean + SD of two independent experiments.

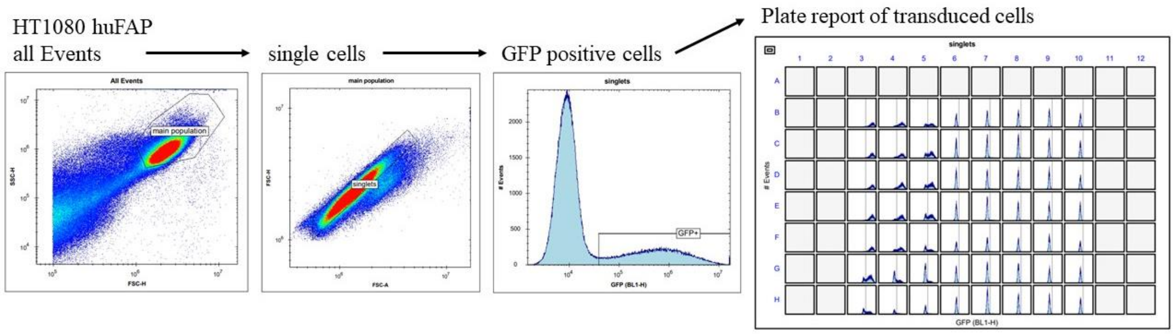

Figure 5. Exemplary overview of the flow cytometry gating strategie of HT1080 huFAP cells after transduction with a titration of rAAV-2E3.v6 and KiH-2E3-MO36. Cells were gated for GFP positive single cells. Flow cytometry and data analysis was performed using the iQue® Screener PLUS (IntelliCyt Cooperation) and ForeCyt® Software (IntelliCyt Cooperation).
